# Erroneous selection of a non-target item improves subsequent target identification in rapid serial visual presentations

**DOI:** 10.2478/v10053-008-0075-3

**Published:** 2010-02-28

**Authors:** Yuki Yamada, Atsunori Ariga, Kayo Miura, Takahiro Kawabe

**Affiliations:** 1 Faculty of Human-Environment Studies, Kyushu University, Fukuoka, Japan; 2 Department of Psychology, The University of Tokyo, Japan

**Keywords:** attentional blink, RSVP, category, attentional set, lag-1 sparing

## Abstract

The second of two targets (T2) embedded in a rapid serial visual presentation (RSVSVP) is often missed even though the first (T1) is correctly reported (attentional blink). The rate of correct T2 identification is quite high, however, when T2 comes immediately after T1 (lag-1 sparing). This study investigated whether and how non-target items induce lag-1 sparing. One T1 and two T2s comprising letters were inserted in distractors comprising symbols in each of two synchronised RSVSVPs. A digit (dummy) was presented with T1 in another stream. Lag-1 sparing occurred even at the location where the dummy was present (Experiment 1). This distractor-induced sparing effect was also obtained even when a Japanese katakana character (Experiment 2) was used as the dummy. The sparing effect was, however, severely weakened when symbols (Experiment 3) and Hebrew letters (Experiment 4) served as the dummy. Our findings suggest a tentative hypothesis that attentional set for item nameability is meta-categorically created and adopted to the dummy only when the dummy is nameable.

## Introduction

Our cognitive processing has severe temporal limitations. For example, *attentional blink* (AB; Raymond, Shapiro, & Arnell, 1992) refers to the phenomenon that occurs when two targets are sequentially embedded in a rapid serial visual presentation (RSVP) of distractors. The identification rate of the subsequent target (T2) is impaired, whereas that of the preceding target (T1) is high. More specifically, T2 performance is substantially impaired when the temporal lag between T1 and T2 is short (or within 500 ms), but recovers for longer lags (e.g., Chun & Potter, 1995; Shapiro, Arnell, & Raymond, 1997; Visser, Zuvic, Bischof, & Di Lollo, 1999).

The AB deficit has been explained in terms of a temporal shortage of attentional resources available for the processing of T2. For example, Shapiro et al. (1997) claimed that the T2 impairment is caused because T1 processing exhausts attentional resources, resulting in a scarcity of resources for T2 processing when the temporal lag between the targets is short. When the resources occupied by T1 are released after the T1 processing is completed, T2 performance recovers from the AB deficit as the temporal lag between the targets increases. Similarly, Chun and Potter (1995) proposed an AB model with two processing stages. In the first stage, parallel processing, all stimuli presented in RSVP are rapidly analysed in a capacity-unlimited manner. The representation at this stage is labile. Next, the serial processing stage consolidates the target to be explicitly reported in a capacity-limited manner. More specifically, it is assumed that the consolidation in the second stage is limited to only one target at a time, and it requires a certain period of processing to complete the consolidation. Hence, whereas the second stage is occupied with T1, the processing for T2 is put off. Consequently, during the consolidation of T1, T2 is forgotten, given that the representation of T2 in the first stage is interfered with by incoming stimuli, resulting in AB.

Under specific conditions, the AB deficit can be avoided. In particular, the T2 identification rate is relatively high when it appears immediately after T1, within about 100 ms (*lag-1 sparing*; Bowman & Wyble, 2007; Chun & Potter, 1995; Hommel & Akyürek, 2005; Martin & Shapiro, 2008; Potter, Chun, Banks, & Muckenhoupt, 1998; Potter, Staub, & O’Connor, 2002; Raymond et al., 1992). In several resource depletion models, it is predicted that the most severe impairment of T2 identification should be observed in the shortest temporal lag. Lag-1 sparing, however, is a phenomenon that contradicts this view. Therefore, the temporary resource depletion for T2 processing alone cannot explain lag-1 sparing. Rather, other factors must also underlie the occurrence of lag-1 sparing.

Di Lollo, Kawahara, Ghorashi, and Enns (2005) have offered an explanation of AB and lag-1 sparing (see also Kawahara, Kumada, & Di Lollo, 2006). In their study, three successive targets, which were letters (T1, T2, and T3), were embedded in an RSVP stream of distractors (e.g., digits). The AB deficit was not observed. In other words, not only lag-1 sparing (for T2) but also lag-2 sparing (for T3) was observed. As lag-2 sparing was not observed when T2 was replaced by a distractor, it was suggested that the category of the item after T1 is critical for the successful selection of a trailing target. Di Lollo et al. explained their results with the notion of *temporary loss of control* (TLC) of the attentional set that accepts task-relevant items (targets) and rejects task-irrelevant items (distractors). That is, only an item that matches the attentional set can be processed further. In the TLC model, it is assumed that the observers initially adopt the attentional set for a target category in an endogenous manner. The attentional set requires periodic maintenance signals from the central executive in the higher brain regions (Kawahara, Kumada, & Di Lollo, 2006). While processing T1 in an RSVP, the central executive loses control and fails to send the signal to maintain the attentional set. The attentional set is easily altered by the intervention of an irrelevant item between T1 and T2, and hence, if the task-irrelevant items appear while the cognitive system is processing T1, AB occurs (Allport, Styles, & Hsieh, 1994; Kawahara, Kumada, & Di Lollo, 2006). If not, the attentional set for the target survives for some lags. This elicits lag-1, lag-2, and lag-3 sparings (Olivers, van der Stigchel, & Hulleman, 2007).

Lag-1 sparing concurrently occurs at multiple locations when attentional set is configured at those locations (Kawahara & Yamada, 2006). These researchers used four alphabetical targets embedded two at a time in two synchronised RSVP streams of distractor digits at the left and right of the centre of the display: T2s appeared concurrently with a variable lag after T1s, which also appeared concurrently. The observers were asked to judge whether the T1s were the same or different and to identify the T2s. As a result, lag-1 sparing concurrently occurred in both streams. Moreover, lag-1 sparing did not occur when two T2s spatially shifted inward, rejecting the possibility that the attentional set encompassed a large area, including the location of both streams. Therefore, Kawahara and Yamada concluded that the cognitive system establishes two split attentional sets at two non-contiguous spatial locations concurrently.

Furthermore, lag-1 sparing occurs even when T1 and T2 categories are different (e.g., Vogel, Luck, & Shapiro, 1998; Yamada & Kawahara, 2007). Yamada and Kawahara used four targets in two RSVP streams. In each stream, two targets were chosen from two target categories (i.e., alphabet letters and Arabic digits) and were inserted into distractors that otherwise comprised two categories (i.e., Japanese katakana characters and pseudo-characters). Consequently, lag-1 sparing occurred even though there was no time for the switching of the attentional set from one category to another. Therefore, they considered the multidimensional attentional set for the two categories to be simultaneously configured at different locations.

In this study, we aimed at further elaborating the hypothetical idea of multidimensional attentional setting, and to this end we tested whether an item in a non-target category affected lag-1 sparing of a trailing target. In previous studies (Kawahara & Yamada, 2006; Yamada & Kawahara, 2007), lag-1 sparing occurred where identification of the item preceding T2 was required. Hence, the occurrence of lag-1 sparing seemed to stem from multidimensional filtering based on a matching between two rapidly detected target categories and the attentional set for each category. On the other hand, the present study examined whether lag-1 sparing was governed by a preceding item that should be ignored. We employed dual-RSVP streams as in previous studies (Kawahara & Yamada, 2006; Potter et al., 2002; Yamada & Kawahara, 2007), but the streams contained only three targets (a single T1 and two T2s). A non-target item was put on the T1 frame in another stream of T1 (we called this item *dummy T1*). That is, one stream had T1 and T2, and the other had the dummy T1 and T2. In this situation, the dummy T1 category should be configured as one of the distractor categories. Moreover, previous studies suggested that lag-1 sparing did not occur when T1 and T2 locations were different (Juola, Botella, & Palacios, 2004; Peterson & Juola, 2000; Visser et al., 1999; Yamada & Kawahara, 2005). Therefore, if filtering relies on strict category matching, lag-1 sparing should not occur in the stream where the dummy T1 is presented.

## Experiment 1

### Method

#### Observers

Ten students from Kyushu University who were unaware of the purpose of the experiment participated. All of them reported normal or corrected-to-normal eyesight.

#### Apparatus and Stimuli

Stimuli were displayed on a 19-inch CRT monitor (EIZO FlexScan T761, Japan) with a resolution of 1024 × 768 pixels and a vertical refresh rate of 75 Hz. A viewing distance of 60 cm was maintained with a head-and-chin rest. A PC/AT-compatible computer controlled the presentation of stimuli and collection of data. Stimuli and experiments were programmed in Delphi 6 (Borland Software Corporation). In every trial, from a set of letters of the English alphabet excluding “I”, “O”, “Q”, and “Z”, three uppercase letters, all different, were randomly chosen as targets. Ten keyboard symbols served as distractors (“!”, “>”, “#”, “<”, “%”, “@”, “?”, “=”, “*”, and “-“). The dummy T1 was an Arabic digit. Each item subtended a visual angle of around 1° in height. The luminance of these items was 2.5 cd/m2 against a background with a luminance of 98.5 cd/m2. The stimulus display comprised a fixation cross at the centre of the screen and two synchronised RSVP streams to the left and right of the fixation cross. T1 was one of the three targets that appeared in one of the two streams, and the T2s of the remaining two targets were simultaneously presented in both streams. The dummy T1 was presented simultaneously with T1 but in another stream. In a trial in which the dummy T1 was absent, a distractor item (i.e., a symbol) was inserted instead. The dummy presence/absence was equally probable. The centre-to-centre distance between the two streams subtended a visual angle of 3.4°.

#### Procedure and Design

**Figure 1.**
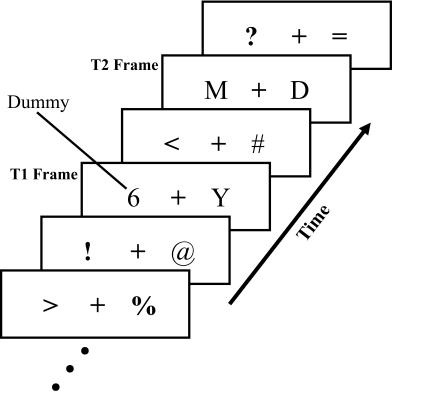
Schematic representation of stimuli in Experiment 1. The letters were targets, and the symbols were distractors. The dummy T1 of digits coincided with the actual T1.

The observers were individually tested in a dark room. Figure 1 illustrates the flow of the experimental trial. After the observers pressed the space bar, two synchronised RSVP streams were presented, containing 8 to 12 leading distractors before the T1 frame. Each item in the streams was displayed for 80 ms, and the inter-stimulus interval (ISI) was 27 ms. In a given trial, the distractors in each stream were randomly selected from a set of symbols, with the constraint that the selected character differed from the immediately preceding one. Moreover, in a given frame, the distractors in both streams differed from each other. T2s appeared after T1— simultaneously in both streams (the T2 frame) — with any one of five lags (107, 214, 320, 427, or 533 ms). The RSVP stream of distractors continued to be displayed during the lag. The T2 frame was followed by one frame of distractors in each stream. The observers identified the three targets and reported them by typing the corresponding keys in no particular order. They were also told that the digits were not the target and had to be ignored. There were 20 practice trials prior to the 200 experimental trials. The experimental session was comprised of three independent variables: presence versus absence of the dummy T1; T1 location (right or left); and lags 1, 2, 3, 4, or 5 (× 107 ms). Each condition was repeated ten times. The trials were conducted in a pseudo-randomised order between the obser-vers.

### Results

**Figure 2.**
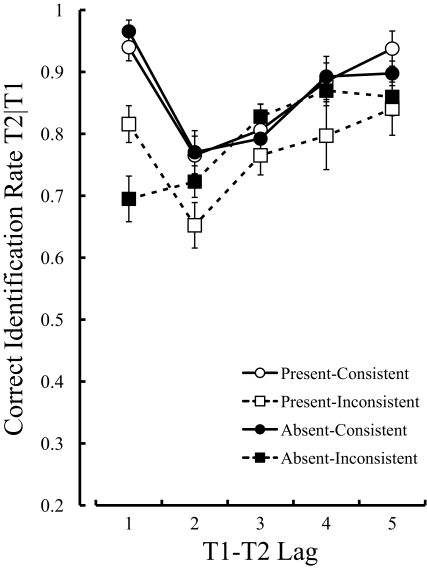
Mean percentage of correct identification of the second targets, given the correct identification of the first targets in Experiment 1. Error bars indicate standard errors.

Figure 2 shows the percentage of correct identification of T2 in each stream when T1 was correctly reported. The rate of correct identification of T1, averaged across all lags, was 81.3%. Because two T2s were presented simultaneously on separate streams in a given trial, each T2 performance when T1 identification was correct was analysed separately. Thus, in this and the subsequent experiments, three factors were the subject of the analysis. The first factor was the presence or absence of the dummy item within RSVP streams (the Dummy factor) to assess how the performance of T2 identification varied with the presence/absence of the dummy item. The second factor was consistency or inconsistency of the locations of T1 and T2 (the T2 location factor) to assess how the performance of T2 identification varied depending on whether the actual T1 and T2 were presented in the same or different streams. The third was five steps (or three steps in Experiment 4) of the inter-target lag (the Lag factor) to assess how the performance of T2 identification varied depending on temporal T2 positions. Moreover, in this and subsequent experiments, lag-1 sparing was defined as the case in which T2 performance at lag 1 was significantly higher than that at lag 2.^1^ Moreover, lag-1 sparing was collaterally defined as the case in which T2 performance at lag 1 was significantly higher in the present condition than in the absent condition.

A three-way analysis of variance (ANOVA) on T2 performance with three within-subject factors showed significant main effects of T2 location, *F*(1, 9) = 14.3, *MSE* = 226.8, *p* < .005, and Lag, *F*(4, 36) = 16.3, *MSE* = 97.0, *p* < .0001. It also revealed significant interactions between Dummy and Lag, *F*(4, 36) = 2.8, *MSE* = 49.91, *p* = .04, between T2 location and Lag, *F*(4, 36) = 7.5, *MSE* = 68.77, *p* = .0002, and among the three factors, *F*(4, 36) = 2.7, *MSE* = 83.18, *p* = .04. The main effect of Dummy, however, was not significant, *F*(1, 9) = 0.6, *p* = .46 Moreover, the interaction between Dummy and T2 location was not significant, *F*(1, 9) = 0.6, *p* = .48 The tests of the simple effects, based on the significant interaction among the three factors, revealed significant simple-simple main effects of Lag in the present-consistent condition, *F*(4, 144) = 8.3, *p* < .0001, present-inconsistent condition, *F*(4, 144) = 7.2, *p* < .0001, absent-consistent condition, *F*(4, 144) = 8.8, *p* < .0001, and absent-inconsistent condition, *F*(4, 144) = 8.7, *p* < .0001. Moreover, a simple-simple main effect of Dummy was found in the inconsistent condition at lag 1, *F*(1, 90) = 10.0, *p* = .002.

Multiple comparisons using Ryan’s method (Ryan, 1960),^2^ based on the simple-simple main effect of Lag, indicated that the correct identification rate of T2 at lag 1 was significantly higher than that at lag 2 in the present-consistent, present-inconsistent, and absent-consistent conditions, *t*(144) = 4.52, *p* < .0001; *t*(144) = 4.23, *p* < .0001; *t*(144) = 5.05, *p* < .0001, respectively. The identification rate of T2 at lag 1, however, was not significantly different from that at lag 2 in the absent-inconsistent condition, *t*(144) = 0.72, *p* = .47.

Additionally, the correct rate for T1 identification was analysed to confirm competition between T1 and the dummy item. A two-tailed *t*-test revealed that the correct rate for T1 identification was significantly lower when T2 appeared at lag 1 in the present condition than when it appeared in the absent condition, *t*(9) = 3.04, *p* = .01.

### Discussion

In Experiment 1, we found that lag-1 sparing was observed for both T2s concurrently. Specifically, lag-1 sparing occurred at a location different from the T1 location only when the dummy T1 was presented. On the other hand, at the T1 location, robust lag-1 sparing occurred regardless of the presence/absence of the dummy item. At the location different from the T1 location, lag-1 sparing did not occur when the dummy T1 was not presented, consistent with previous studies showing that lag-1 sparing occurred only when a common location was shared by T1 and T2 (Juola et al., 2004; Peterson & Juola, 2000; Visser et al., 1999; Yamada & Kawahara, 2005). The competition between T1 and the dummy item suggested that the dummy item was involuntarily processed.

Additionally, performance at a location different from the T1 location was severely impaired at lag 1 when the dummy item was absent, even though performance at the T1 location was quite high. These results support the notion that the T2 item in each stream was processed as a part of a discrete attentional episode established at each stimulus location. That is, each stream of RSVP seems to be filtered by an attentional set that can be split into multiple locations and works independently (Kawahara & Yamada, 2006; Yamada & Kawahara, 2007).

Not all the results of this experiment, however, can be explained by multidimensional attentional setting (Yamada & Kawahara, 2007) based on the TLC model (Di Lollo et al., 2005). In Experiment 1, letters and symbols were used as targets and distractors, respectively, and digits were used as the dummy T1. According to TLC, the observers’ attentional set should not have been multidimensional but adopted only for letters because digits were not the target. In TLC, because category matching between an attentional set and an item category is fundamentally the mechanism of input filtering, digits should have been considered as distractors to be ignored and consequently altered attentional setting for targets (letters) if they came up during T1 processing. This would predict a severe T2 deficit (i.e., AB) at lag 1, not lag-1 sparing. Lag-1 sparing, however, clearly occurred only after presentation of the dummy T1. Therefore, filtering by attentional set based on TLC category matching cannot explain the results. An alternative mechanism for simultaneous processing of multiple categories, other than multidimensional attention setting, should be postulated.

One might argue that the results of Experiment 1 reflect the adoption of an attentional set configured for an alphanumeric category, which is a meta-category of letters and digits. That is, it was possible that the observers in Experiment 1 adopted the attentional set that corresponds to a category including both letters and digits all together, namely, alpha-numerals. Thus, an alphanumeric attentional set might be applied to both the dummy and T2, resulting in conventional lag-1 sparing. The next experiment examined this possibility by introducing a new category, which is not included in the alphanumeric category, as a dummy category.

## Experiment 2

This experiment aimed at testing whether the adoption of an alphanumeric attentional setting produced lag-1 sparing as in the first experiment. In Experiment 2, a new category, Japanese katakana, was used as the category of the dummy T1. This category was quite familiar to the Japanese observers employed in this experiment and was not included in the alphanumeric category. If the results of Experiment 1 were a product of alphanumeric attentional setting, lag-1 sparing should not occur even when the dummy T1 of Japanese katakana was presented.

### Method

#### Observers

Eleven Japanese students from Kyushu University, including one of the authors (Y.Y.), participated in this experiment. Except for Y.Y., they were unaware of the purpose of the experiment. All of them reported normal or corrected-to-normal eyesight.

#### Apparatus, Stimuli, and Procedure

The apparatus, stimuli, and procedure were identical to those in Experiment 1 except that, instead of digits, 10 Japanese katakana characters, “ア” (a), “イ” (i), “ウ” (u), “エ” (e), “オ” (o), “カ” (ka), “キ” (ki), “ク” (ku), “ケ” (ke), and “コ” (ko), were introduced as dummies. The observers were asked to ignore Japanese katakana.

### Results

**Figure 3.**
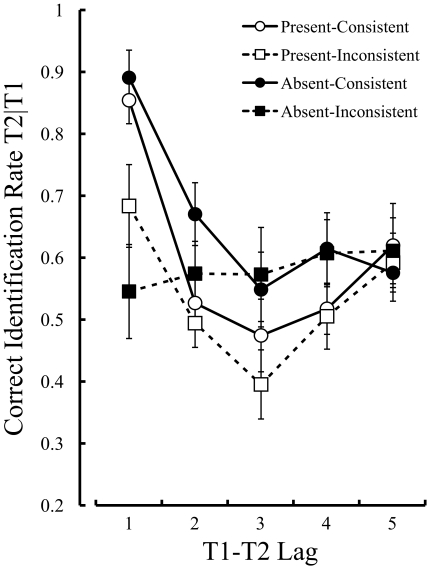
Mean percentage of correct identification of the second targets, given the correct identification of the first targets in Experiment 2. Error bars indicate standard errors.

Figure 3 shows the percentage of correct identification of T2 in each stream when T1 was correctly reported. The correct identification of T1, averaged across all lags, was 64.9%. A three-way ANOVA on T2 performance with three within-subject factors (Dummy: present or absent, T2 location: consistent or inconsistent, Lag: 1–5) showed significant main effects of Dummy, *F*(1, 10) = 11.5, *MSE* = 143.03, *p* = .007, T2 location, *F*(1, 10) = 10.0, *MSE* = 276.52, *p* = .01, and Lag, *F*(4, 40) = 11.3, *MSE* = 327.02, *p* < .0001. Significant interactions between Dummy and Lag, *F*(4, 40) = 4.4, *MSE* = 161.61, *p* = .005, between T2 location and Lag, *F*(4, 40) = 6.2, *MSE* = 205.49, *p* = .0006, and among the three factors, *F*(4, 40) = 2.9, *MSE* = 114.42, p = .03, were obtained. The interaction between Dummy and T2 location, *F*(1, 10) = 0.1, *p* = .80, was not significant. Tests of the simple effects, based on significant interactions among the three factors, revealed significant simple-simple main effects of Lag in the present-consistent condition, *F*(4, 160) = 12.7, *p* < .0001, present-inconsistent condition, *F*(4, 160) = 6.4, *p* = .0001, and absent-consistent condition, *F*(4, 160) = 10.2, *p* < .0001, but not in the absent-inconsistent condition, *F*(4, 160) = .40, *p* = .81. Moreover, simple-simple main effects of Dummy were found in the inconsistent condition at lag 1, *F*(1, 100) = 6.6, *p* = .01, and at lag 3, *F*(1, 100) = 10.9, *p* = .001.

Multiple comparison tests using Ryan’s method, based on the simple-simple main effect of Lag, indicated that the correct identification rate of T2 at lag 1 was significantly higher than that at lag 2 in the present-consistent condition, *t*(160) = 5.40, *p* < .0001, present-inconsistent condition, *t*(160) = 3.12, *p* = .002, and absent-consistent condition, *t*(160) = 3.64, *p* = .0004. A post hoc *t*-test did not reveal a significant difference between the performances at lag 1 and lag 2 in the absent-inconsistent condition, *t*(10) = 0.44, *p* = .67.

A two-tailed *t*-test did not reveal any difference between the correct identification rates of T1 when T2 appeared at lag 1 in the present condition and when T2 appeared in the absent condition, *t*(10) = 0.13, *p* = .90.

### Discussion

In this experiment, as well as in Experiment 1, lag-1 sparing with Japanese katakana as the dummy T1 was clearly observed. The involvement of an alphanumeric attentional setting for both letters and digits can still explain lag-1 sparing observed in Experiment 1, but cannot explain the results of Experiment 2.

Why did lag-1 sparing occur even though no attentional set was configured for the dummy T1? As a straightforward interpretation suggests, it is likely that the dummy T1 erroneously served as the actual T1, leading to the lag-1 sparing of the trailing T2. In this interpretation, an attentional set for targets or an attentional set for distractors, related to active selection or active rejection, would be involved in this erroneous selection. The cognitive system seemed mistakenly to select the dummy T1 because the dummy category (digits or Japanese katakana) was similar to the target category (letters) or because the dummy category was different from the distractor category (symbols) that made up the majority of RSVP streams and consequently was not rejected.

## Experiment 3

This experiment examined whether a dummy item belonging to a category which was simply different from a distractor category led to lag-1 sparing. In Experiment 3, the categories of dummy items and distractors used in Experiment 1 were reversed (i.e., symbols and digits served as dummies and distractors, respectively). Despite the categorical reversal, the dummy and target categories were still clearly separated although the difference between the dummy and distractor categories remained unchanged from that in Experiment 1. Lag-1 sparing would occur when a dummy symbol item was presented if mere categorical difference between the dummy and distractor was the decisive factor.

### Method

#### Observers

Fourteen students from Kyushu University participated, and none of the students were aware of the purpose of the experiment. All reported normal or corrected-to-normal eyesight.

#### Apparatus, Stimuli, and Procedure

The fundamental aspects of the apparatus, stimuli, and procedure were identical to those in Experiment 1, with the following exceptions: The dummy T1 category was changed to symbols, and the category of the distractors was changed to digits. The observers were asked to ignore the symbols.

### Results

**Figure 4.**
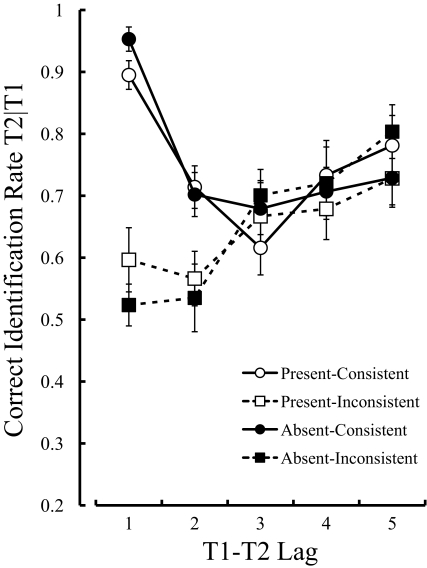
Mean percentage of correct identification of the second targets, given the correct identification of the first targets in Experiment 3. Error bars indicate standard errors.

Figure 4 shows the correct identification rate for T2 in each stream when T1 was correctly reported. The correct identification rate of T1, averaged across all lags, was 83.7%. A three-way ANOVA on T2 performance with three within-subject factors (Dummy: present or absent, T2 location: consistent or inconsistent, Lag: 1–5) showed significant main effects of T2 location, *F*(1, 13) = 16.9, *MSE* = 405.87, *p* = .001, and Lag, *F*(4, 52) = 7.2, *MSE* = 226.64, *p* = .0001. It also revealed significant interactions between T2 location and Lag, *F*(4, 52) = 28.0, *MSE* = 137.69, *p* < .0001, and among the three factors, *F*(4, 52) = 3.9, *MSE* = 87.7, *p* = .007. The main effect of Dummy was not significant, *F*(1, 13) = 0.4, p = .53. Moreover, the interactions between Dummy and T2 location, *F*(1, 13) = 0.02, *p* = .90, and between Dummy and Lag, *F*(4, 52) = 1.2, *p* = .33, were not significant. Tests of the simple effects, based on the significant interaction among the three factors, revealed significant simple-simple main effects of Lag in the present-consistent condition, *F*(4, 208) = 10.9, *p* < .0001, present-inconsistent condition, *F*(4, 208) = 4.5, *p* = .002, absent-consistent condition, *F*(4, 208) = 13.3, *p* < .0001, and absent-inconsistent condition, *F*(4, 208) = 15.8, *p* < .0001. Moreover, simple-simple main effects of Dummy were found in the inconsistent condition at lag 1, *F*(1, 130) = 4.2, *p* = .04, and lag 5, *F*(1, 130) = 4.5, *p* = .04.

Multiple comparisons using Ryan’s method, based on the simple-simple main effect of Lag, indicated that the correct identification rate of T2 at lag 1 was significantly higher than that at lag 2 in the present-consistent condition, *t*(208) = 4.15, *p* < .0001, and the absent-consistent condition, *t*(208) = 5.75, *p* < .0001. The difference between the correct identification rate of T2 at lag 1 and at lag 2, however, did not reach significance in the present-inconsistent condition, *t*(208) = 0.70, *p* = .49, or absent-inconsistent condition, *t*(208) = 0.26, *p* = .79. That is, lag-1 sparing was not observed in these inconsistent conditions.

A two-tailed *t*-test did not reveal any difference between the correct identification rates of T1 when T2 appeared at lag 1 in the present condition and when T2 appeared in the absent condition, *t*(13) = 0.14, *p* = .89.

### Discussion

In this experiment, lag-1 sparing was attenuated even when the dummy T1 was present. Specifically, although T2 performance at lag 1 was not significantly higher than that at lag 2 in the inconsistent condition, T2 performance at lag 1 in the Dummy-present condition was higher than in the Dummy-absent condition. Moreover, the analysis of T1 performance suggests that the dummy T1 symbol did not impair the performance for the actual T1. These results suggest that lag-1 sparing largely depends on potential common properties between the dummy and target categories rather than on the categorical difference between the dummy and distractor categories.

What then was the common property of the dummy and target categories producing lag-1 sparing in this study? A tentative answer to this question is the nameability of items. Names of items were different among the categories used in the previous experiments. For example, an item belonging to letter, digit, and Japanese katakana categories is easy to name. Such easy-to-name dummy items might have produced lag-1 sparing in Experiments 1 and 2. Symbols such as “$”, “#”, and “!”, however, are difficult to name. The difficult-to-name dummy items might not have produced lag-1 sparing in Experiment 3. If easy-to-name items were preferentially treated by the cognitive system, the attentional set would be erroneously adopted for such items, resulting in lag-1 sparing.

## Experiment 4

Experiment 4 was performed to determine whether lag-1 sparing with the dummy items depended on item nameability. We used Hebrew alphabet letters as dummy categories, Roman alphabet letters and symbols as target categories, and digits as the distractor category. Data were collected from Japanese students who knew the shape of Hebrew alphabet letters, but could not name an individual letter. If item nameability underlies dummy-driven lag-1 sparing, no lag-1 sparing with the dummy item from Hebrew letters would be observed because Japanese participants could not name them.

### Method

#### Observers

Twelve Japanese adults participated in this experiment. All of them were unaware of the purpose of the experiment and reported normal or corrected-to-normal eyesight.

#### Apparatus, Stimuli, and Procedure

This experiment was similar to Experiment 1 except for the following. First, categories of targets, dummies, and distractors were changed. Ten Roman alphabet letters (“A” to “K” excluding “I”) or 10 symbols used in the previous experiments were employed as the targets. Digits served as the distractors. Ten Hebrew alphabet letters, “א” (alef), “ב” (bet), “ג” (gimel), “ד” (dalet), “ה” (he), “ז” (zayin), “ח” (chet), “ט” (tet), “ל” (lamed), and “ש” (shin), were introduced as dummies. A pre-experiment questionnaire revealed that none of the observers knew Hebrew at all, and the observers were asked to ignore the Hebrew letters within the RSVP streams. Second, only lags 1, 2, and 5 were used. Thus, each observer performed 120 trials with two experimental blocks including two target-category conditions (Roman alphabet or symbol). Each block contained 2 dummy conditions (present or absent) × 2 T1 location conditions (right or left) × 3 lag conditions (lag 1, 2, or 5) × 5 replications. In each block, the trial order was randomised. The order of the blocks was counterbalanced across observers.

### Results

**Figure 5.**
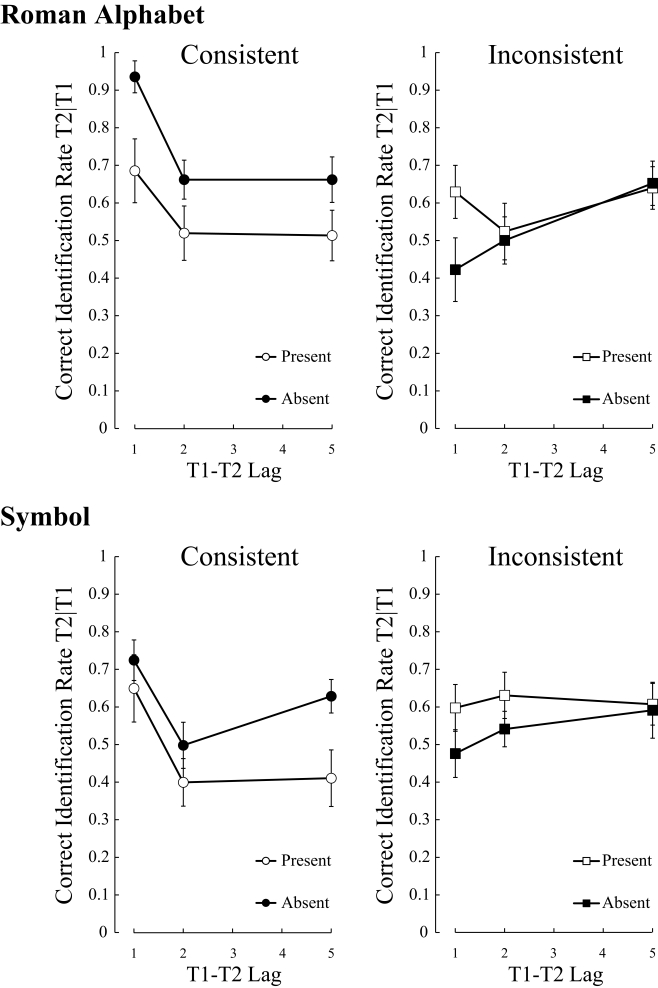
Mean percentage of correct identification of the second targets, given the correct identification of the first targets in Experiment 4. The upper panels and lower panels represent the results of the Roman alphabet and symbol conditions, respectively. The left panels and right panels represent the results of the consistent and inconsistent conditions, respectively. Error bars indicate standard errors.

Figure 5 shows the results of Experiment 4. The correct identifications of T1, averaged across all lags, in the Roman alphabet and symbol conditions were 71.1 % and 69.9 %, respectively. The results of the Roman alphabet and symbol conditions were analysed separately.

#### Roman alphabet condition.

A three-way ANOVA on T2 performance with three within-subject factors (Dummy: present or absent, T2 location: consistent or inconsistent, Lag: 1, 2, or 5) showed a significant main effect of Lag, *F*(2, 22) = 4.3, *MSE* = 383.17, *p* = .03. It also revealed significant interactions between Dummy and T2 location, *F*(1, 11) = 14.3, *MSE* = = 402.29, *p* = .003, between T2 location and Lag, *F*(2, 22) = 9.3, *MSE* = 382.93, *p* = .001, and among the three factors, *F*(2, 22) = 4.1, *MSE* = 227.80, *p* = .03. The main effects of Dummy, *F*(1, 11) = 4.7, *p* = .05, and T2 location, *F*(1, 11) = 3.5, *p* = .09, were marginally significant. An interaction between Dummy and Lag, *F*(2, 22) = 0.2, *p* = .79, was not significant. Tests of simple effects based on the interaction between Dummy and T2 location revealed a significant simple main effect of Dummy in the consistent condition, *F*(1, 22) = 18.8, *p* = .0003. Tests of the simple effects based on the significant interaction among the three factors revealed significant simple-simple main effects of Lag in the present-consistent condition, *F*(2, 88) = 3.2, *p* = .05, absent-consistent condition, *F*(2, 88) = 8.3, *p* = .0005, and absent-inconsistent condition, *F*(2, 88) = 4.6, *p* = .01, but not in the present-inconsistent condition, *F*(2, 88) = 1.4, *p* = .26. Multiple comparisons using Ryan’s method, based on the simple-simple main effect of Lag, indicated that the correct identification rate of T2 at lag 1 was no different from that at lag 2 in the absent-inconsistent condition, *t*(88) = 1.00, *p* = .32. A post hoc *t*-test did not reveal a significant difference in the performance at lag 1 and lag 2 in the present-inconsistent condition, *t*(11) = 1.20, *p* = .25. Moreover, a significant simple-simple main effect of Dummy was acknowledged in the inconsistent condition at lag 1, *F*(1, 66) = 7.8, *p* = .007. Additionally, a *t*-test revealed that T1 performance was significantly lower when T2 appeared in the present-consistent condition than when it appeared in the absent-consistent condition, *t*(11) = 2.32, *p* = .04.

#### Symbol condition

Because a three-way ANOVA on T2 performance with three within-subject factors did not show a significant interaction among the three factors, *F*(2, 22) = 0.02, *p* = .98, separate one-way ANOVAs on T2 performance with Lag as a factor were performed. As a result, significant main effects in the present-consistent condition, *F*(2, 22) = 3.7, *p* = .04, and absent-consistent condition, *F*(2, 22) = 4.4, *p* = .02, were found. The main effects, however, in the absent-inconsistent condition, *F*(2, 22) = 0.1, *p* = .93, and present-inconsistent condition, *F*(2, 22) = 1.0, *p* = .38, were not significant. Post hoc *t*-tests did not reveal a significant difference in the performance at lag 1 and lag 2 in the present-inconsistent condition, *t*(11) = 0.33, *p* = .75, and absent-inconsistent condition, *t*(11) = 0.73, *p* = .48. Moreover, the difference in the performance at lag 1 between the present and absent conditions was not significant, *t*(11) = 1.17, *p* = .27. Furthermore, T2 performance averaged across lags in the absent-consistent condition was marginally significantly higher than that in the present-consistent condition, *t*(11) = 2.16, *p* = .05. Additionally, a *t*-test revealed that T1 performance was significantly lower when T2 appeared in the present-consistent condition than when it appeared in the absent-consistent condition, *t*(11) = 2.66, *p* = .02.

### Discussion

The results showed that lag-1 sparing with the dummy item was weakened in the Roman alphabet condition and disappeared in the symbol condition when a Hebrew alphabet letter, which was not nameable by the Japanese observers who participated in this experiment, was employed as the dummy item. The results are consistent with the prediction that item nameability strongly influences dummy-driven lag-1 sparing. This idea is compatible with the present results in that weak or no sparing effect was found in this experiment because Hebrew and symbols were not nameable. Moreover, the results in the symbol condition suggest that a mere categorical difference between the dummy and distractor categories does not determine lag-1 sparing.

An unexpected finding in this experiment was that T2 performance in the stream consistent with the actual T1 dropped when the dummy item was presented. This was not a general tendency in the previous experiments in this study. Hence, this finding seems to be a stimulus-specific one. In Experiment 4, Hebrew alphabet characters were employed as dummy items, and Japanese observers did not know these characters. We surmise that quite unfamiliar items like Hebrew characters increased overall processing cost, affecting processing of the actual T1 and trailing T2. This is beyond the scope of the present study, but we may examine this issue in future research.

## General Discussion

The present study found that a non-target item in neither a target nor distractor category can elicit lag-1 sparing. Experiment 1 showed that a dummy T1 (digits) not belonging to a target category (Roman alphabet) produced lag-1 sparing. Moreover, in Experiment 2, it was demonstrated that a dummy item from Japanese katakana caused lag-1 sparing for the following T2 of Roman alphabet letters, suggesting that dummy-based lag-1 sparing occurs beyond an alphanumeric attentional setting. Additionally, Experiment 3 showed that a dummy item from symbols did not cause robust lag-1 sparing, suggesting that the mere presence of the dummy item at the temporal location of T1 does not explain dummy-based lag-1 sparing. Finally, the results of Experiment 4 suggest that nameability of the dummy item was related to dummy-driven lag-1 sparing. Categories such as the Roman alphabet, Japanese katakana letters, and digits were nameable whereas symbols and the Hebrew alphabet were not nameable by observers in the present experiments. Our findings suggest that attentional set for item nameability is meta-categorically created and adopted to the dummy T1 only when the dummy T1 is nameable. The idea of a meta-categorical attentional set for nameability is consistent with almost all the results in this study.

**Figure 6.**
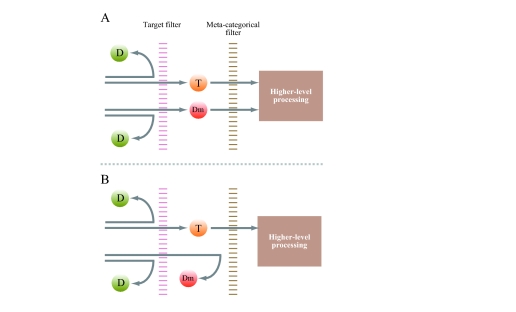
Hypothetical two-stage filtering. The cases of a dummy of nameable items (A) and a dummy of symbols (B) are shown. T = Target, D = Distractor, Dm = Dummy. In the first stage, the target filter selects potential targets, and in the second stage, the meta-categorical filter selects nameable items from the outputs of the first filter.

How can the cognitive system differentiate target from distractors if the meta-categorical attentional set is actually adopted? Simple assumptions about the meta-categorical attentional setting for nameability cannot explain why in Experiment 4 the observers could differentiate letter targets from the digit distractors. Here we assume two-stage filtering, as illustrated in Figure 6. At the first filtering, the explicit distractors are eliminated. Hence, the potential targets are passed towards higher-level processing. At the second filtering, items are discriminated in terms of whether they are nameable. The second filtering corresponds to the meta-categorical attentional setting that we are now proposing.

The results of a previous study (Yamada & Kawahara, 2007) might also be relevant to the meta-categorical attentional setting. The investigators demonstrated that simultaneous lag-1 sparings occurred at the right and left RSVP streams even when the two target categories introduced into the RSVPs consisted of two distractor categories (Japanese katakana and pseudocharacters). Given our findings, their results possibly stemmed from a meta-categorical attentional set for alphanumeric or nameable items that handles two target categories together.

One might argue that the present results stemmed from an artefact involving a low-level visual feature of the stimuli used in the experiments. Maki, Bussard, Lopez, and Digby (2003) showed that symbols are significantly different from Roman alphabet letters and digits in terms of pixel density. The difference in pixel density among categories might serve as the subject of adoption of attentional setting. That is, the similarity in pixel density between the dummy T1 and T2 might have been higher in Experiment 1 (i.e., dummy T1 of digits) than in Experiment 3 (i.e., dummy T1 of symbols), leading to the presence of lag-1 sparing in the former but to its absence in the latter. To clarify this issue, we calculated the number of pixels per character (as pixel density) for the five categories used in the present study. The mean pixel density of the Roman alphabet, Hebrew alphabet, digits, Japanese katakana, and symbols was 666.0 (*SD* = 115.8), 421.7 (*SD* = 100.5), 545.9 (*SD* = 105.0), 711.5 (*SD* = 98.8), and 479.9 (*SD* = 388.2), respectively. The results of statistical comparisons^3^ ruled out the possibility that the similarity of pixel density between the dummy T1 and T2 underlay lag-1 sparing based on the dummy T1. Despite no significant difference in pixel density between digits and symbols, lag-1 sparing was present with dummy digits in Experiment 1; it was absent with dummy symbols in Experiment 3. Therefore, it is unlikely that pixel density explains the dummy-induced sparing effect.

An account based on feature dissimilarity between the dummy and distractors may, however, explain the present results that cannot be explained by item nameability. These results were the higher T2 performance at lag 1 in the dummy-present condition compared with that in the dummy-absent condition in Experiment 3 and the similar difference in the Roman alphabet condition of Experiment 4. Maki et al. (2003) showed that symbols are most distinctive in visual features among letters, digits, symbols, and false font characters. It is probable that the Hebrew letters as well as symbols may have had feature dissimilarity from digits that were used as distractors in Experiments 3 and 4. We surmise that the dummy T1 with visual features dissimilar from distractors was admitted to higher-level processing without altering the attentional set for nameability, resulting in weak lag-1 sparing of a trailing target. Since, however, lag-1 sparing vanished when the target category was symbols (Experiment 4), lag-1 sparing with the dummy T1 dissimilar from distractors might be limited to the condition in which target categories were nameable.

Other than the filtering dependent on attentional set, an attentional mechanism may explain the lag-1 sparing with the dummy item. In a previous study, Potter et al. (2002) suggested with the two-stage competition model that attention is labile at the first stage until an initially processed target has been consolidated at the second stage. If a potential target comes along during this period, it attracts the attentional resources necessary for processing the initial target. In the present study, the dummy item was simultaneously presented with the actual T1. Hence, it is likely that the dummy item attracted some attention during T1 processing because attention was labile in this period. Moreover, a recent study showed that transient attention was triggered by a categorically defined target (e.g., a letter or digit), and the attentional enhancement provided a benefit for the subsequent target processing in a short period, about 100 ms (Wyble, Bowman, & Potter, 2009). In the present study, T2 at the dummy location (i.e., in the inconsistent condition) might have profited from transient attentional enhancement owing to the dummy that attracted attention during the actual T1 processing. Furthermore, Wyble and co-workers speculated that the categorical difference between targets and distractors contributed to the targets’ ability to trigger transient attention. This speculation and our findings may closely converge on the following point: At least unnameable items cannot trigger enough transient attention to bring benefits to T2 at the same location.

A meta-categorical setting is not an irrational idea. Previous studies have suggested that the character style or the type style is also the subject of an attentional set. For example, reported findings show that an attentional set was adopted for uppercase words inserted in the RSVP of lowercase words (Broadbent & Broadbent, 1987). Additionally, an item written in a typewriter font is processed with an attentional set differently from an item written in a script font (Kawahara, Enns, & Di Lollo, 2006). Consequently, the cognitive system flexibly tunes an attentional set to various properties of characters. We suggest that the meta-categorical attentional setting for nameability can be considered similar to these attentional sets tuned to character/type style. Exploring the relationship between the limit of setting (e.g., the range of categorical levels or the number of categories) and its effect on attentional processes (e.g., the required resource or time) may be an issue for future research. To this end, a cognitive linguistic approach may also be required.

## Footnotes

1 Previous studies on AB have simultaneously employed three indices to measure lag-1 sparing more sensitively. The first index was the superior T2 performance at lag 1 to that at lag 2 (e.g., Potter et al., 2002; Yamada & Kawahara, 2007). The second was the superior T1 performance at lag 1 to that at lag 2 (e.g., Potter et al., 2002; Hommel & Akyürek, 2005). The third was the higher proportion of reversal for reported temporal order between T1 and T2 at lag 1 in comparison with that at other lags (e.g., Bowman & Wyble, 2007; Chun & Potter, 1995). In the present study, however, it seemed plausible to adopt only the first index to assess lag-1 sparing because we used anomalous stimuli with a asymmetrical number of items between T1 and T2 (i.e., one T1 and two simultaneous T2s) different from those used in canonical AB studies with symmetrical number of items between T1 and T2. Hence, the source of the difference in T1 performance across conditions was hard to specify because, in our experiments, T1 performance seemed to be influenced from the processing of the dummy T1, T2 in the T1 stream, and T2 in another stream simultaneously. Hence, we did not use the second index. Furthermore, the third index was not considered as an effective index because two factors seemed to be confounded: the necessity of reporting both T2s and the presence of dummy items.

2 Ryan’s method adopts nominal significance level α’ given as follows: α’ = 2α / [*n* × (*m* – 1)], where α means whole significance level, *n* means the number of groups to be compared, and m means the distance defined as the number of groups X_p_ satisfying X_i_ ≤ X_p_ ≤ X_j_. Here, X_i_ and X_j_ are a pair in a concerned hypothesis. The degrees of freedom are given as *N* – *n*, where *N* means sample size.

We performed a between-subjects one-way ANOVA on the pixel density. A significant main effect was found, *F*(4, 45) = 3.5, *p* = .02. However, multiple comparisons using Ryan’s method revealed that the pixel density was significantly different only between Hebrew alphabet letters and Japanese katakana, *t*(45) = 3.1, *p* = .003. A post hoc Welch’s t test revealed that the pixel density of symbols was not different from that of digits, *t*(10) = 0.5, *p* = .63.

## Appendix A

### Supplementary analysis

It would be of interest to know whether the attentional setting for nameable items shares mechanisms involved in reading. Japanese participants in this study read words from left to right; hence, we assume that this mechanism would be most likely to select nameable characters arising at the right of T1. In line with this idea, lag-1 sparing with the dummy item would be more pronounced, perhaps even restricted to cases in which the dummy item appears to the right of T1. Thus, we additionally analysed the left-right asymmetry in the effect of the dummy item with one-way ANOVAs with Lag as a factor. The data from the present-inconsistent condition in Experiments 1 and 2 was the subject of the analysis because these experiments showed strong lag-1 sparing, and hence these data were more likely to show asymmetric lag-1 sparing corresponding to the reading direction. The results showed that main effects of Lag were found in the left stream in Experiment 1, *F*(4, 36) = 8.2, *p* = .0001, and in the left stream, *F*(4, 40) = 3.8, *p* = .01, and right stream, *F*(4, 40) = 2.6, *p* = .05, in Experiment 2. Moreover, a significant difference between lag-1 and lag-2 in the left stream in Experiment 1 (*p* < .0001) was found as well as a significant difference between lag-1 and lag-3 in the left and right streams in Experiment 2 (ps < .005). No main effect, however, was obtained in the right stream in Experiment 1. These results suggest that, in contrast to our prediction, no systematic asymmetry in lag-1 sparing corresponding to the reading direction occurred. If anything, a left-stream advantage possibly exists. Although the present results did not demonstrate an asymmetric effect based on the reading direction, this issue deserves to be examined further. For example, we are interested in a comparison between performances of observers in cultures with left-to-right reading direction (e.g., Japanese or English speakers) and right-to-left reading direction (e.g., Arabic or Hebrew speakers) when the dummy category is or is not nameable.
